# Construction of a Novel Mitochondria-Associated Gene Model for Assessing ESCC Immune Microenvironment and Predicting Survival

**DOI:** 10.4014/jmb.2310.10052

**Published:** 2024-02-22

**Authors:** Xiu Wang, Zhenhu Zhang, Yamin Shi, Wenjuan Zhang, Chongyi Su, Dong Wang

**Affiliations:** 1 Department of General Practice, Shandong Provincial Hospital Affiliated to Shandong First Medical University, Jinan 250021, P.R. China; 2 Department of Thoracic Surgery, Shandong Provincial Hospital Affiliated to Shandong First Medical University, Jinan 250021, P.R. China; 3 School of Foreign Languages, Shandong University of Finance and Economics, Jinan 250014, P. R. China; 4 Department of Surgical, Shandong Provincial Hospital Affiliated to Shandong First Medical University, Jinan 250021, P.R. China; 5 Department of Emergency, Shandong Provincial Hospital Affiliated to Shandong First Medical University, Jinan, 250021, P.R. China

**Keywords:** Esophageal squamous cell carcinoma, weighted gene co-expression network analysis, mitochondria, immune microenvironment, drug susceptibility

## Abstract

Esophageal squamous cell carcinoma (ESCC) is among the most common malignant tumors of the digestive tract, with the sixth highest fatality rate worldwide. The ESCC-related dataset, GSE20347, was downloaded from the Gene Expression Omnibus (GEO) database, and weighted gene co-expression network analysis was performed to identify genes that are highly correlated with ESCC. A total of 91 transcriptome expression profiles and their corresponding clinical information were obtained from The Cancer Genome Atlas database. A mitochondria-associated risk (MAR) model was constructed using the least absolute shrinkage and selection operator Cox regression analysis and validated using GSE161533. The tumor microenvironment and drug sensitivity were explored using the MAR model. Finally, in vitro experiments were performed to analyze the effects of hub genes on the proliferation and invasion abilities of ESCC cells. To confirm the predictive ability of the MAR model, we constructed a prognostic model and assessed its predictive accuracy. The MAR model revealed substantial differences in immune infiltration and tumor microenvironment characteristics between high- and low-risk populations and a substantial correlation between the risk scores and some common immunological checkpoints. AZD1332 and AZD7762 were more effective for patients in the low-risk group, whereas Entinostat, Nilotinib, Ruxolutinib, and Wnt.c59 were more effective for patients in the high-risk group. Knockdown of TYMS significantly inhibited the proliferation and invasive ability of ESCC cells in vitro. Overall, our MAR model provides stable and reliable results and may be used as a prognostic biomarker for personalized treatment of patients with ESCC.

## Introduction

Esophageal cancer (EC) is a common malignancy ranking seventh in incidence and sixth in its fatality rate worldwide [1]. EC is characterized by a poor prognosis, with 880,000 EC-related deaths and 960,000 new EC cases expected each year worldwide by 2040 [2]. The primary histological type of EC is esophageal squamous cell carcinoma (ESCC), which has a very poor prognosis, with a 5-year survival and metastasis rates of 20% and 5% for EC and ESCC, respectively. Optimization of therapeutic regimens and the discovery of safe and effective drugs are important for ESCC treatment.

Mitochondria produce adenosine triphosphate and regulate the generation and clearance of reactive oxygen species. Based on a growing body of evidence, mitochondria play an important role in regulating cellular functions, metabolism, cell growth, and cell death during carcinogenesis [3]. Previous studies have shown that inhibition of mitochondrial metabolism can block tumor progression. Signal transducer and activator of transcription 3β interferes with the mitochondrial electron transport chain, thereby enhancing the chemosensitivity of ESCC [4]. Therefore, reliable mitochondria-related biomarkers are needed to predict ESCC prognosis.

Although relevant models have been developed to predict the survival of patients with ESCC [5, 6], a mitochondria-related ESCC prognostic model has not been constructed. Tumor growth, invasion, and metastasis are influenced by the tumor microenvironment (TME), which also affects the response of patients to treatment [7]. Despite a growing understanding of tumor cell-TME interactions, detailed study of the biological significance of different cells within the TME in ESCC is lacking. The immunological checkpoint blockade response and TME constitution are closely connected [8, 9]. To examine the relationship between risk scores and the TME, we explored TME features, immune cell infiltration in the TME, and immune checkpoint expression levels. To predict the prognosis and determine the immunotherapy response of patients with ESCC, we constructed a mitochondria-associated risk (MAR) model. This model should serve as a reliable prognostic biomarker for personalized treatment of patients with ESCC.

## Materials and Methods

### Data Downloading

The ESCC datasets (GSE20347[10] and GSE161533[11]), were downloaded from the Gene Expression Omnibus (GEO) database (http://www.ncbi.nlm.nih.gov/geo/). The GSE20347 dataset was used for weighted gene co-expression network analysis (WGCNA) and differential gene analyses, while the GSE161533 dataset was used for validation. The ESCC dataset were downloaded from TCGA (https://portal.gdc.cancer.gov/) using the ‘TCGAbiolinks’ package in R software [12]. A total of 91 ESCC samples were obtained for further analysis, and models with no prognosis and duplicate data were eliminated (Table S1). The mitochondria-associated genes were identified via a search of the literature [13] (Table S2).

### WGCNA

WGCNA is an algorithm used to assess gene expression patterns in large numbers of samples [14]. Gene co-expression modules in the tumor and normal groups were developed using WGCNA. First, we calculated the variance of each gene expression value and filtered out genes with an absolute deviation >25%. We constructed scale-free networks using a soft threshold β of 12, with *p* < 0.05 defined as significant. Pearson correlation analysis was performed to determine the correlation between the modules and the disease phenotype of interest, with darker colors indicating stronger correlations. Genes from modules with the highest correlations were selected for further analysis.

### Acquisition of Differentially Expressed Mitochondria-Associated Genes

The tumor and normal groups were established using samples in the GSE20347 dataset. Differential expression analysis was conducted using the R package, ‘limma’ [15], and volcano plots were generated by using ‘ggplot2’ [16]. The differentially expressed genes (DEGs) were identified using the criteria: |log2(FC)| > 1 and Adjusted *p* < 0.05. Venn diagrams were generated to identify the intersecting differentially expressed mitochondria-associated genes (DEMAGs) between DEGs, WGCNA-related genes, and mitochondria-related genes.

### Enrichment Analysis of Differentially Expressed Mitochondria-Associated Genes

The Search Tool for the Retrieval of Interacting Genes/Proteins (STRING) database (http://cn.string-db.org) was used to conduct protein-protein interaction (PPI) analysis of DEMAGs. The PPI network was visualized using Cytoscape software (version 3.8.2). The cytoHubba plugin (version 0.1) in Cytoscape was used to identify the regulatory node proteins. Gene ontology (GO) and Kyoto Encyclopedia of Genes and Genomes (KEGG) pathway analyses were also performed. GO analysis included biological processes (BP), cellular components (CC), and molecular functions (MF).

### Prognostic Marker Screening

The least absolute shrinkage and selection operator (LASSO) is a biased estimation that considers data with complex covariance [17]. Forest maps were generated based on the results obtained for mitochondria-related differences. Univariate Cox regression and LASSO regression analyses were performed to identify prognostic indicators and genes associated with survival in patients with ESCC.

### Risk score Construction and Prognostic Prediction Modeling

The risk score (RS) was calculated using the following formula:

$$\text{Risk score =}\sum_{i=0}^{n}Coef_{i}\times Exp_{i}$$

The regression analysis coefficient (Coef), gene expression value (Exp), and number of genes (n) were used in the calculations. To plot the survival curves, patients were divided into high- and low-risk groups according to their median risk score, and survival at 1, 2, and 3 years was predicted using the ‘survivalROC’ package [18]. Thereafter, the ROC curve was plotted and the area under curve (AUC) values were calculated. The ‘rms’ package [19] was used to display the nomograms and calibration curves of the multifactorial model, and the C-index was calculated to predict survival

### ESTIMATE Immunoreactivity Analysis

Tumor immunoreactivity was assessed using the ‘ESTIMATE’ package in R [20]. Estimation of STromal and Immune cells in MAlignant Tumor tissues using expression data (ESTIMATE) mainly uses the transcriptional patterns of cancer samples to infer the content of tumor cells and infiltrating immune and stromal cells, thereby revealing the ratio or abundance of stromal, immune, and tumor cells related to the TME in the tumor tissue. The immunological and stromal scores of each patient were calculated to compare score differences between the high-and low-risk groups.

### Immunization Microenvironment Analysis

To compare the immune cell characteristics between the risk groups, the infiltration status of 28 immune cells in the high- and low-risk groups using the ssGSEA function in the ‘gsva’ package were assessed [21]. The correlation between the risk score and immune-infiltrating cells was analyzed using Pearson's correlation analysis (PCA). Cibersortx [22] was used for further validation. As immunotherapy currently plays an essential role in oncology, some common immunological checkpoints were also analyzed.

### Drug Sensitivity Analysis

Genomics of Drug Sensitivity in Cancer (GDSC) [23] is a free database for cancer molecular therapy and mutation exploration. Cell line mutation data and 50% inhibiting concentration (IC_50_) values of anticancer drugs were downloaded using the R package, ‘pRRophetic’ [24]. Finally, the correlation between patients with high and low risk score and different anticancer drug sensitivity was assessed.

### Cell Lines

Three ESCC cell lines (HEEC, KYSE30, and KYSE150) were purchased from the Cell Resource Center of Shanghai Institutes for Life Sciences, Chinese Academy of Sciences (China). All cells were cultured in RPMI-1640 medium (Kibbutz Beit HaEmek, Israel) containing 10% fetal bovine serum at 37°C.

### RNA Extraction and Quantitative RT-PCR

Total RNA was extracted from cells using RNAiso Plus (TaKaRa, Japan) and reverse-transcribed into cDNA using an RT kit (TaKaRa). Quantitative PCR (qPCR) was performed on a LightCycler 480 system using SYBR Green Real-Time PCR Master Mix (TaKaRa); while GAPDH was used as an internal reference gene. Relative expression levels were analyzed using the 2^-ΔΔCt^ method [25]. The primer sequences are listed in Table 1.

### Western Blot Analysis

Proteins were extracted from the cells using RIPA buffer (Nanjing Jiancheng Institute of Biotechnology, China) and protease inhibitor (Nanjing Jiancheng Institute of Biotechnology). Protein samples were prepared using a BCA protein assay kit (Nanjing Jiancheng Institute of Biotechnology), separated using sodium dodecyl sulfate polyacrylamide gel electrophoresis, and transferred onto a polyvinylidene difluoride membrane. The membranes were blocked with 5% milk for 1 h and incubated overnight at 4°C with a primary antibody against ALOX12 and TYMS (1:1,000; Abcam, UK), followed by incubation with horseradish peroxidase-labeled secondary antibodies (1:1,000; Zhongshan Biological Technology Co., Ltd., China) for 1.5 h. The protein bands were detected using chemiluminescence.

### Lentiviral Transfection

TYMS RNA interference and negative control lentiviral vectors were obtained from Genechem (China). The lentiviral vectors were introduced into KYSE150 and KYSE30 cells and screened with medium containing 5 μg/ml puromycin to obtain stably transfected cell lines.

### CCK 8 Assay

KYSE150 cells were seeded into 96-well plates at a density of 5 × 10^4^ cells per well and cell proliferation was evaluated at the four time points (0 h, 24 h, 48 h, and 72 h) using the CCK-8 kit (Solarbio, CA1210) according to the manufacturer’s protocol. Then, the absorbance was measured at the wavelength of 490 nm (Thermo Multiskan Sky, China).

### Wound Healing Assay

KYSE150 cells at a density of 5 × 10^4^ were seeded into a six-well plate and carefully wounded using a yellow pipette tip (200 μl). The cell monolayer was washed three times using phosphate-buffered saline (PBS) to remove cells suspended in the culture medium after scratching. Cells were observed with an inverted microscope at 0 and 48 h.

### Transwell Assay

Matrigel gel (BD, 356234, USA) was diluted in serum-free cell culture medium at a ratio of 1:3 at 4°C (the consumables were pre-cooled and the process was completed on an ice plate (MedChemExpress, China). An amount of 50 μl was added to a Transwell chamber. The samples were left at 37°C for 30 min until the Matrigel had solidified. KYSE150 cells (200 μl, 1.0 × 10^4^ cells per well) resuspended in serum-free medium were added to the upper chamber and 600 μl of complete culture medium were added to the lower chamber. The chamber cells were cleaned with PBS and cells were stained with crystal violet.

### Statistical Analysis

R software (version 4.1.0, http://www.R-project.org) was used for all data processing and analyses. We assessed the normality of the data using the Shapiro–Wilk test. For data that did not conform to a normal distribution, appropriate nonparametric tests were applied. Continuous variables are presented as the mean ± standard deviation. Two sets of continuous variables were compared using the Wilcoxon rank–sum test, and the statistical significance of regularly distributed variables was determined using Student’s *t*-test. Unless indicated to the contrary, the results were assessed using Spearman’s correlation analysis of the correlation coefficients between different molecules. All *p*-values were two-sided, and a *p*-value <0.05 indicated statistical significance.

## Results

### Acquisition of Differentially Expressed Mitochondria-Associated Genes (DEMAGs)

**Fig. 1:**
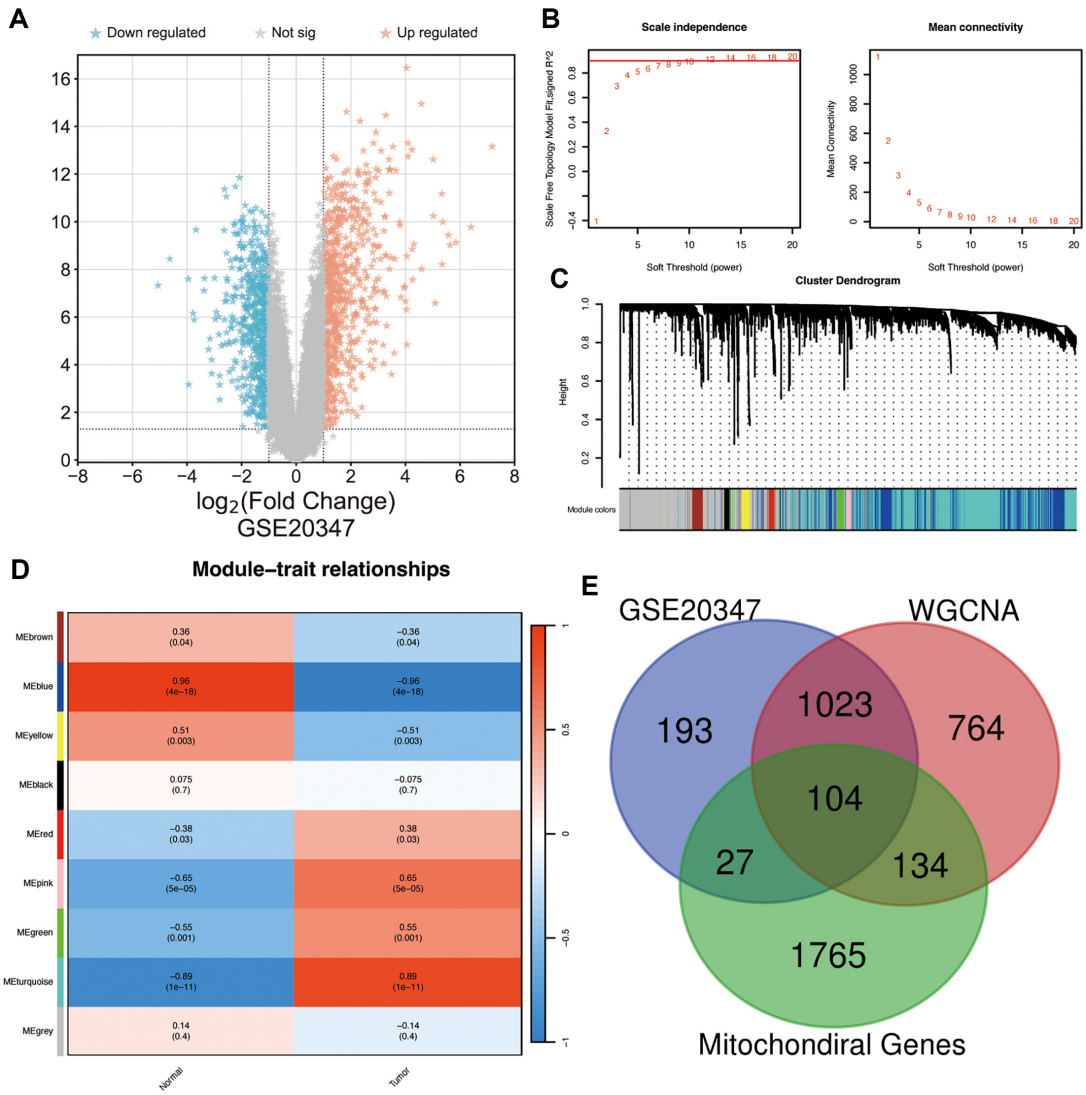
Acquisition of the relevant differentially expressed genes. (**A**) GSE20347 differential gene volcano plot. (**B**) Power parameter screening process of weighted gene co-expression network analysis (**WGCNA**). (**C**) Gene clustering analysis using WGCNA presented as a tree diagram. (**D**) Gene classification modules are depicted in different colors; a darker color indicates a higher degree of correlation; the correlation coefficients and *p*-values are shown in the grids within the matrices. (**E**) Wayne plots of the three parameters.

To identify DEGs between ESCC and paired, non-tumorous controls, GEO2R analysis was performed based on the normalized data from GSE20347. We obtained 1,127 DEGs and generated a volcano plot (Fig. 1A). Using WGCNA, nine modules were identified in GSE20347 (Fig. 1B-C), each of which is represented by a different color (Fig. 1D). The relationships between modules were evaluated using Spearman's correlation coefficients. Two modules, "blue" and "turquoise," were highly correlated with the highest positive and negative correlations of ESCC (blue: r = -0.97, p = 4e-18, genes = 696; turquoise: r = 0.89, *p* < 0.001, genes = 1329). Through overlay analysis of the 1,127 DEGs, nine modules, and 2,030 mitochondria-associated genes, 104 DEMAGs were identified (Fig. 1E).

### Functional Enrichment Analysis

**Fig. 2:**
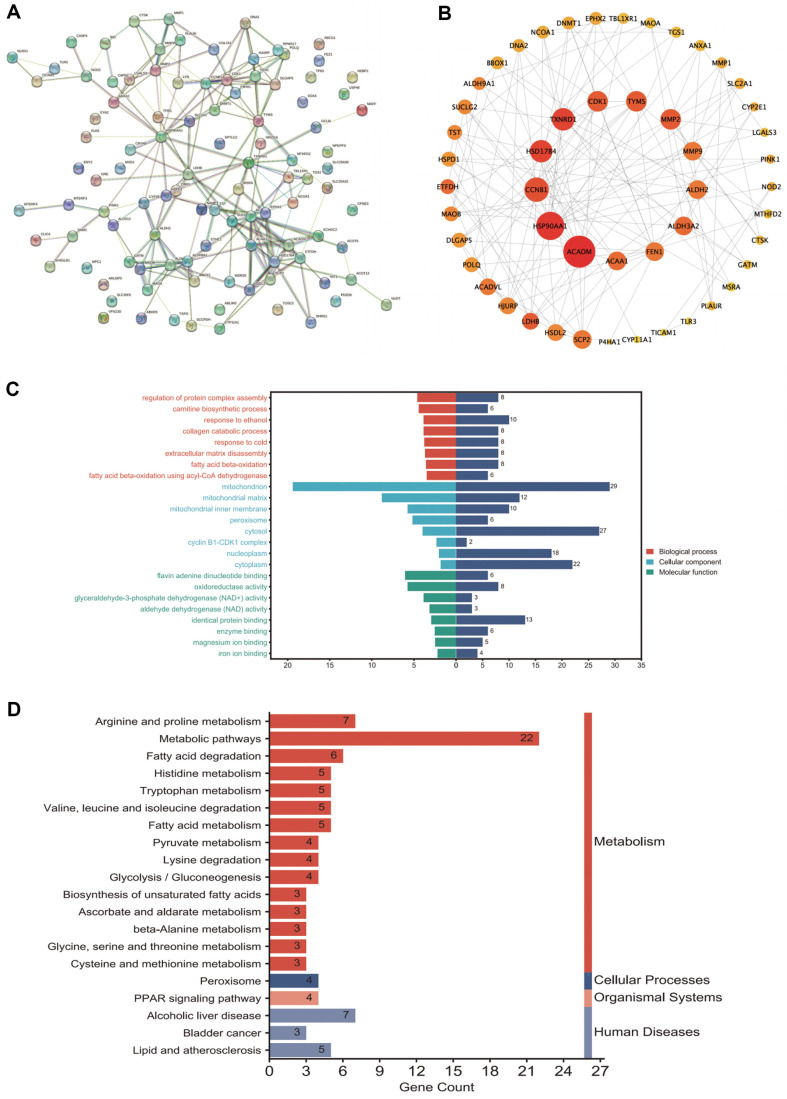
GO-KEGG enrichment analysis of co-DEGs. (**A**) Protein–protein interaction network of co-DEGs. (**B**) Top 50 genes obtained based on the degree parameter. (**C**) The GO enrichment analysis (longer right bars indicate significant enrichment and more enriched genes). (**D**) The KEGG enrichment analysis (longer columns indicate more enriched genes; redder colors indicate more enrichment).

Protein–protein interaction networks of the DEMAGs were constructed using STRING and visualized using Cytoscape (Fig. 2A). The top 50 most connected genes were selected using Degree analysis in the cytoHubba plugin (Fig. 2B). To elucidate the functions of the DEMAGs, we performed functional enrichment analysis. We found that the DEMAGs were substantially enriched in biological processes related to the regulation of protein complex assembly, carnitine biosynthetic processes, response to ethanol, and collagen catabolic processes. In terms of cellular components, DEMAGs were mainly enriched in the mitochondria, mitochondrial matrix, and inner mitochondrial membrane. The DEMAGs were enriched in molecular functions such as flavin adenine dinucleotide binding, oxidoreductase activity, and glyceraldehyde-3-phosphate dehydrogenase activity (Fig. 2C). KEGG enrichment analysis revealed substantial enrichment in metabolic pathways, arginine and proline metabolism, and fatty acid degradation (Fig. 2D).

### Risk Score Construction

**Fig. 3:**
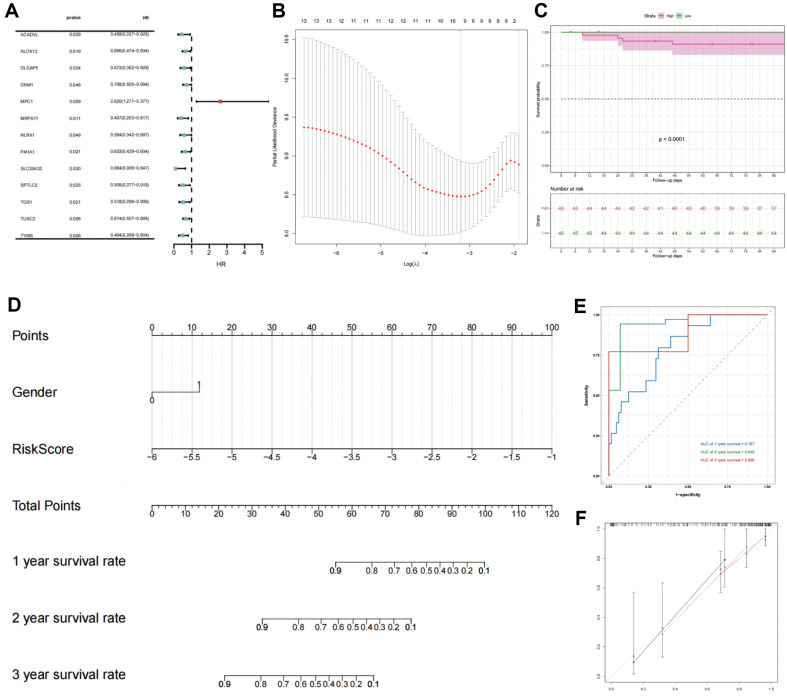
Analysis of differentially expressed prognostic genes. (**A**) Forest plots of differentially expressed mitochondrial genes. (**B**) LASSO regression diagnostic model. (**C**) Kaplan–Meier (K-M) curve. (**D**) Nomogram construction. (**E**) Receiver operating characteristic (**ROC**) curve of nomogram scores. (**F**) Calibration curve of the columnar plots; horizontal coordinates represent survival predicted from the columnar plots, whereas the vertical coordinate represents actual observed survival.

To assess the prognostic relevance of 104 DEMAGs in patients with ESCC, univariate Cox regression analysis was conducted using 91 TCGA-ESCC patients; 13 core genes were identified as significant (ACADVL, ALOX12, DLGAP5, DNM1, MPC1, MRPS17, NLRX1, P4HA1, SLC25A32, SPTLC2, TGS1, TUSC3, and TYMS), and thus may have a strong effect on ESCC prognosis (*p* < 0.05) (Fig. 3A). An ideal lambda value of 10 was obtained in cross-validation analysis (Fig. 3B). Based on the median RS, two groups of patients with ESCC were identified: high-risk (*n* = 45) and low-risk (*n* = 46). The prognostic survival of patients with a high RS was substantially lower than that of patients with a low RS (Fig. 3C).

### Prognostic Prediction Modeling

According to the results of univariate Cox regression analysis (Table S3), in addition to RS, patient survival is also affected by gender. Multifactorial prognostic forest plots (Fig. 3D) were constructed using Cox regression models to predict the model. To verify the prediction accuracy, ROC analysis was performed. The time ROC of the prognostic model showed high predictive capacity for predicting the overall survival of patients with ESCC, with area under the curve values of 0.797, 0.946, and 0.886 for 1, 2, and 3 years, respectively (Fig. 3E). The calibration curve was also plotted (Fig. 3F), revealing a good fit.

### Multifactorial Cox Regression Analysis

Thirteen core genes were validated using the independent external dataset, GSE161533, and the results were consistent with those of our assessment (Fig. S1A). We performed multifactorial Cox regression on the 13 one-factor Cox regressions (Fig. S1B). Two multifactorial, correlated genes, ALOX12 and TYMS (Table 2), were obtained. Survival analysis was conducted for the high- and low-risk groups using ALOX12 and TYMS. The Kaplan–Meier curves revealed *p*-values of 0.053 and 0.0024, respectively (Fig. S1C-S1D). For in-depth evaluation, we also performed ROC analysis (Fig. S1E). The area under the curve values of ALOX12 and TYMS were 0.5958 and 0.6253, respectively. These results indicate that TYMS had a greater impact on the survival of patients than ALOX12 did.

### ESTIMATE Immunoreactivity Assay

**Fig. 4:**
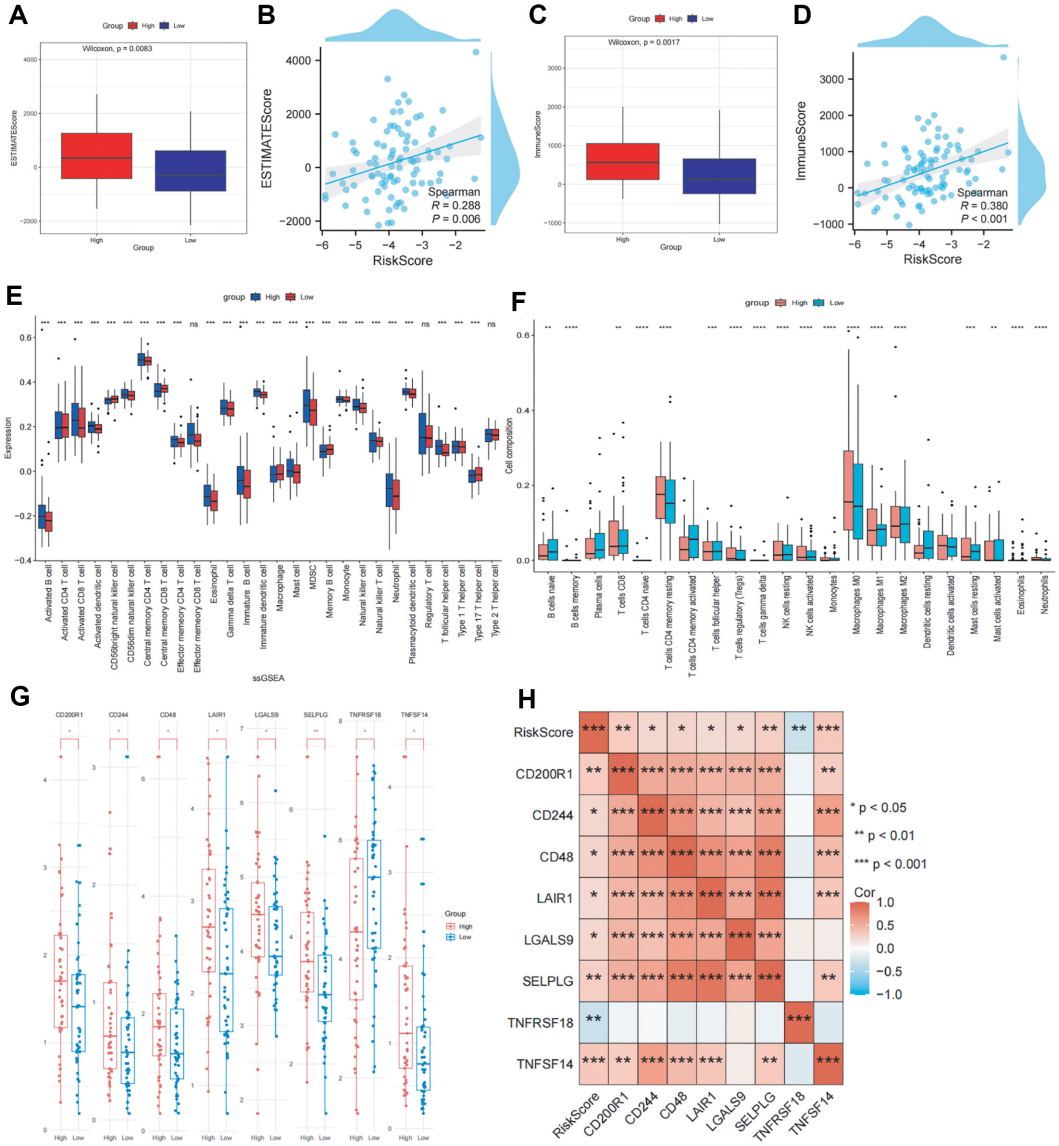
Different immune profiles between the high- and low-risk groups in TCGA–ESCC dataset. (**A**) Difference in ESTIMATEScore between the high-risk and low-risk groups. (**B**) Correlation between ESTIMATEScore and risk scores. (**C**) Difference in immune scores between the high- and low-risk groups. (**D**) Correlation between the immune score and risk score. (**E**) The ssGSEA scores of 28 immune cell types between the high- and low-risk groups. (**F**) CIBERSORT analysis. (**G**) Expression analysis of the immune checkpoints. (**H**) Heat map of the correlation between risk score and immune checkpoints.

The ESTIMATEScore was higher in the high-risk group than in the low-risk group (Fig. 4A). Consistently, a positive correlation was found between RS and the ESTIMATEScore (Fig. 4B). We explored the correlation between RS and the immune microenvironment. In ESCC, the ImmuneScore was also higher in the high-risk than in the low-risk group (Fig. 4C), and RS was positively correlated with the ImmuneScore (Fig. 4D). Collectively, these results suggest that RS is closely related to the TME.

### Immunoassays for the High- and Low-Risk Groups

To further evaluate the effect of RS on the general immunological profile of patients with ESCC, we performed single-sample gene set enrichment analysis (ssGSEA) to infer immune infiltration in the high- and low-risk groups from TCGA–ESCC dataset (Fig. 4E). Using the CIBERSORT package, we examined the invasive ability of several immune cells. The infiltration of 22 immune cells into various subgroups is shown in Fig. 4F. The results of CIBERSORT analysis were comparable to those of ssGSEA, validating our findings. Taken together, these results suggest that the tumor immune microenvironment is related to the poor prognosis of patients with ESCC in the high-risk group; however, further studies are needed to validate this result. Immune checkpoint inhibitors are widely used in tumor immunotherapy. Eight immune checkpoints were differentially expressed between the high-and low- groups (*p* < 0.05) (Fig. 4G). A heat map of the correlation between RS and these immune checkpoints (*p* < 0.05) (Fig. 4H) revealed that RS was correlated with all immune checkpoints.

### Sensitivity Analysis of Drugs for Different Risk Subgroups

**Fig. 5:**
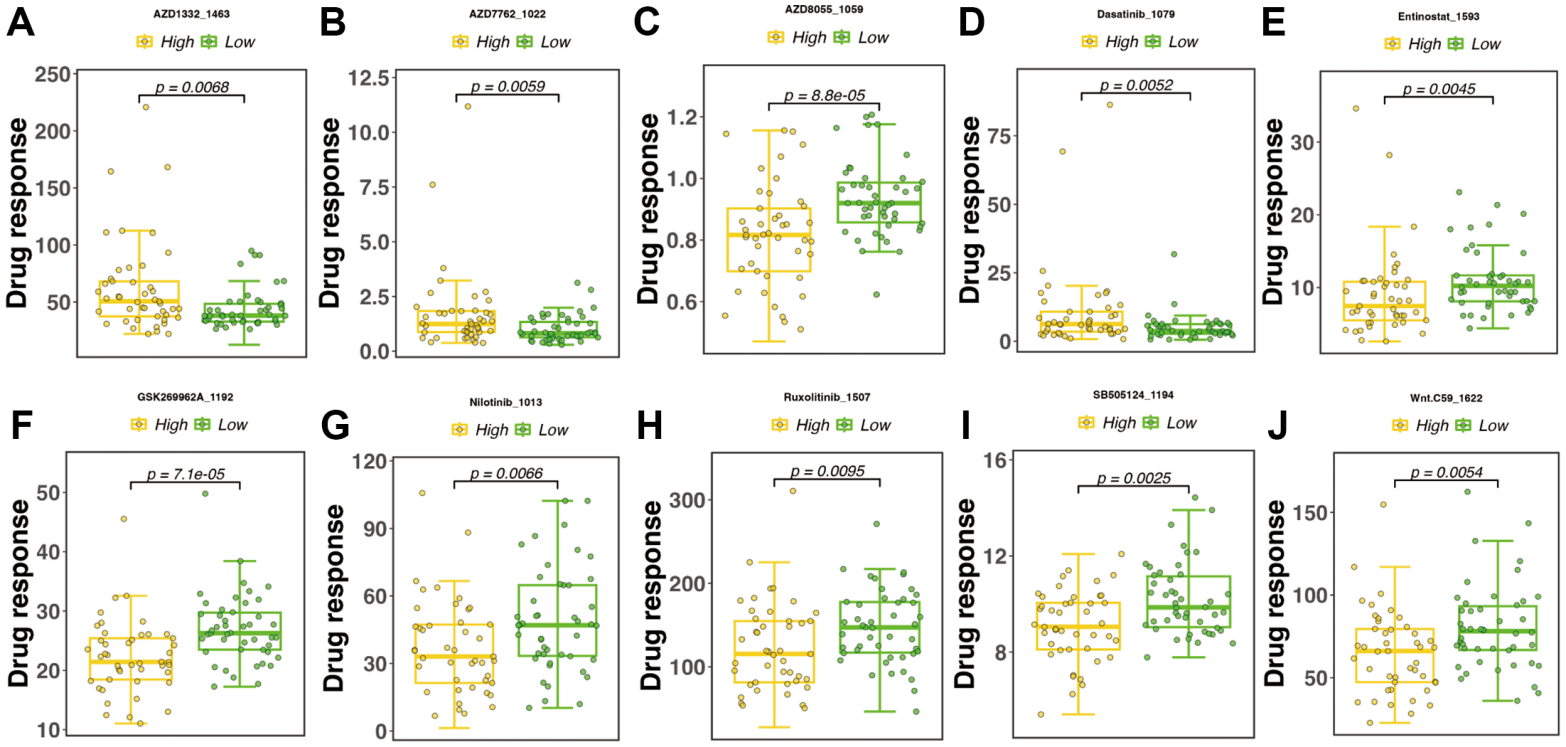
Drug sensitivity analysis. (**A–J**) Top 10 drugs showing the highest sensitivity with risk score.

To determine whether RS can be used as an index for predicting the response to drugs, we inferred the IC_50_ values of 198 drugs in TCGA-ESCC patients. The IC_50_ values differed between the high- and low-risk groups (*p* < 0.01). The top 10 drugs were AZD1332 (Fig. 5A), AZD7762 (Fig. 5B), AZD8055 (Fig. 5C), Dasatinib (Fig. 5D), Entinostat (Fig. 5E), GSK269962A (Fig. 5F), Nilotinib (Fig. 5G), Ruxolutinib (Fig. 5H), SB505124 (Fig. 5I), and Wnt.c59 (Fig. 5J). These 10 drugs were also correlated with TYMS (Fig. S2A–S2J). Information on these top 10 drugs is presented in Table 3.

### Protein Level Expression and RNA Expression in the ESCC Cell Lines

**Fig. 6:**
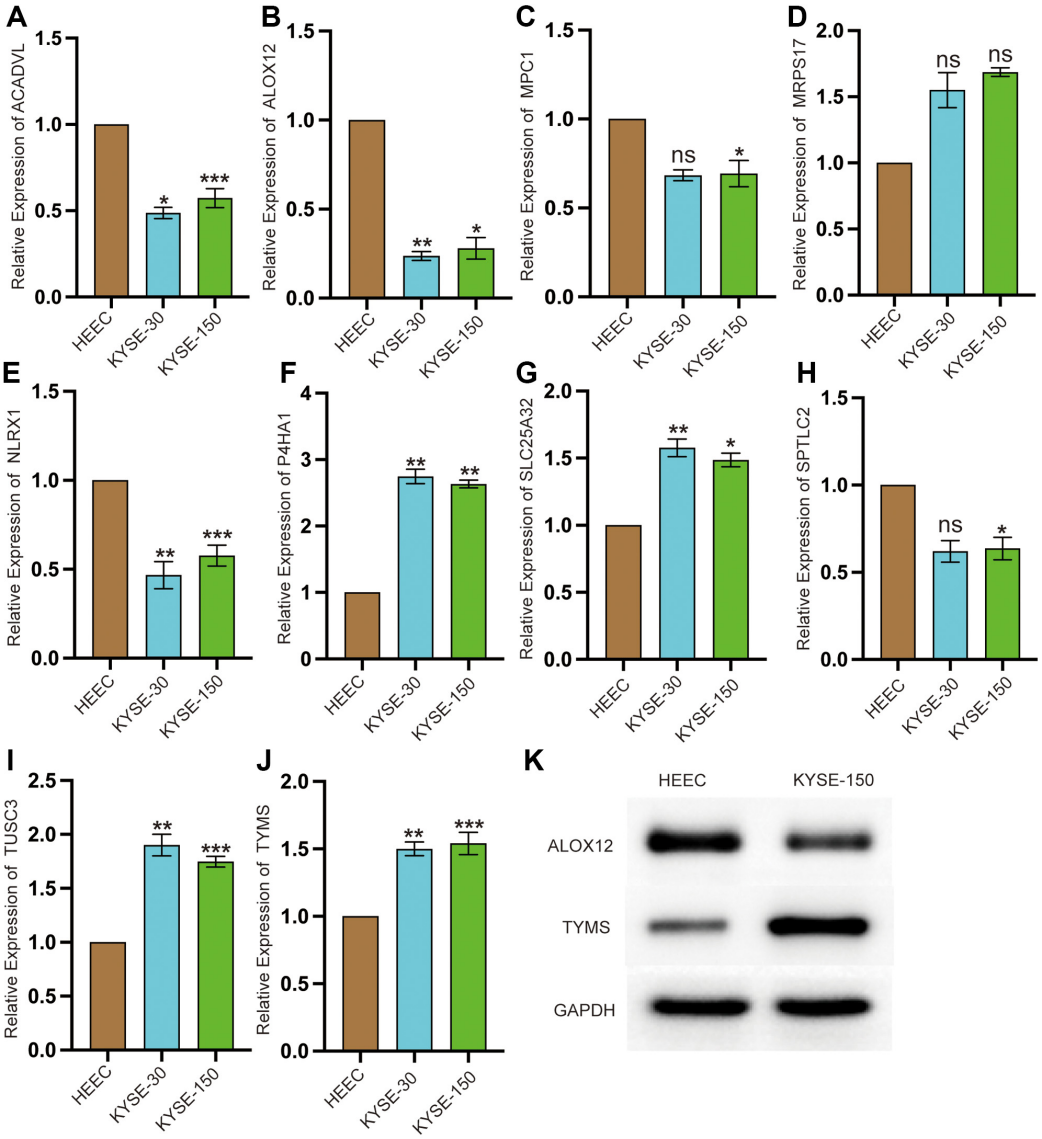
Relative mRNA expression levels of core genes. (**A–J**) Relative mRNA expression levels of ACADVL, ALOX12, MPC1, MRPS17, NLRX1, P4HA1, SLC25A32, SPTLC2, TUSC3, and TYMS were confirmed using quantitative PCR. Data are presented as the mean standard deviation. *p < 0.05, **p < 0.01, ***p < 0.001. (**K**) Protein expression levels of TYMS and ALOX12 in the ESCC cell line and normal cell line. *p < 0.05.

We performed RT-qPCR to access the mRNA expression levels of the hub genes in two ESCC cell lines and one normal ESCC cell line. The expression levels of MRPS17, P4HA1, SLC25A32, TUSC3, and TYMS in the ESCC cell lines were higher than those in the normal cell line (Fig. 6D, 6F, 6G, 6I, and 6J; *p* < 0.05). The expression levels of ACADVL, ALOX12, MPC1, NLRX1, and SPTLC2 were lower in the ESCC cell lines than those in the normal cell line (Fig. 6A–6C, 6E, and 6H; *p* < 0.05). Western blot analysis revealed that the protein expression of TYMS in the ESCC cell line was higher than that in the normal cell line. The protein expression of ALOX12 in the ESCC cell line was lower than that in the normal cell line (Fig. 6K, *p* < 0.05).

### TYMS Promotes ESCC Cell Proliferation

**Fig. 7:**
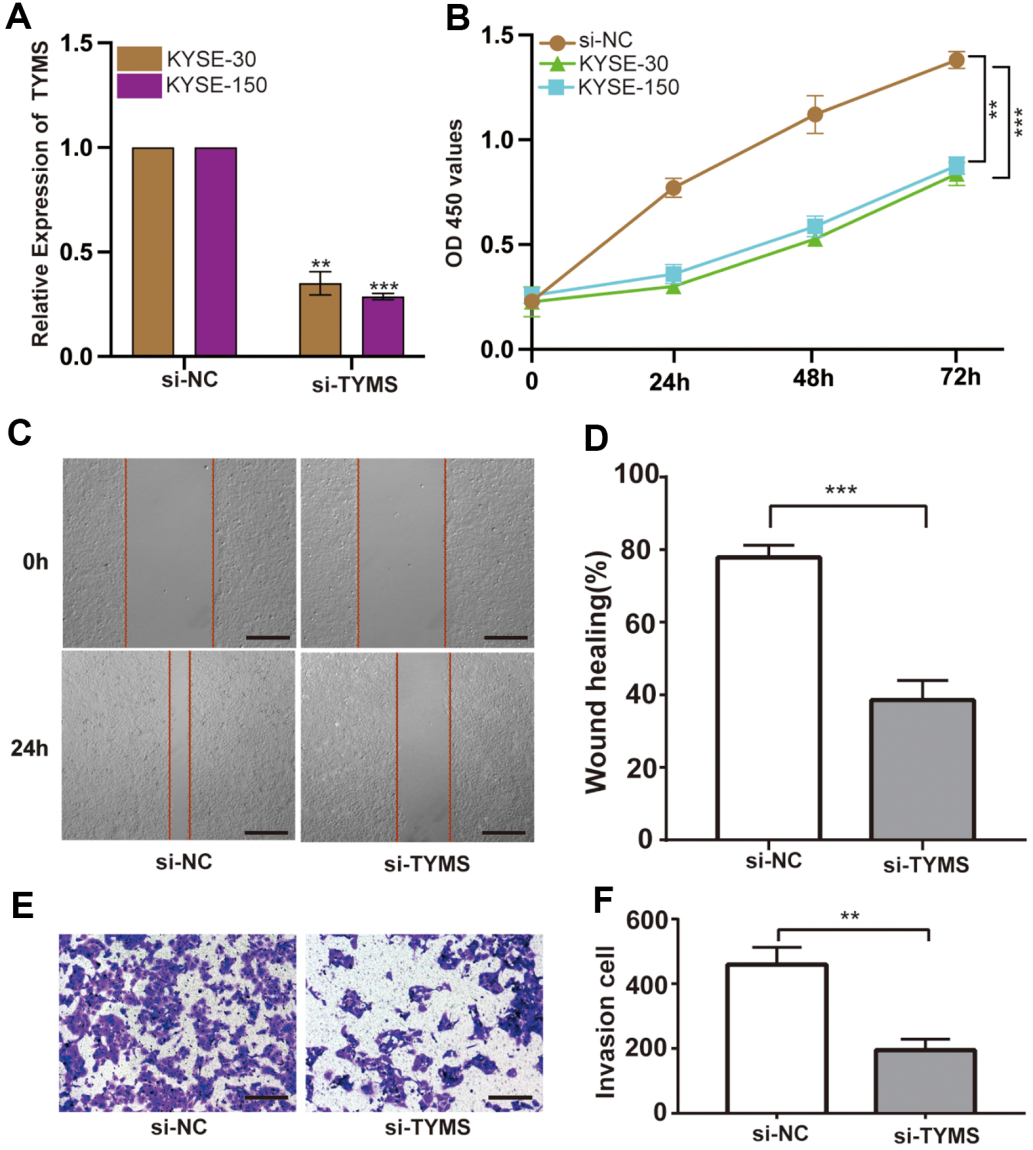
TYMS promotes the proliferation of ESCC cells. (**A**) Efficiency of the transfection of the lentiviral vectors. (**B**) CCK8 assay to assess the proliferation abilities of cells from the different subgroups. (**C-D**) Wound healing assay to detect the wound-healing abilities of the cells from the different treatment groups. (**E-F**) TYMS knockdown suppresses the proliferation ability of ESCC cells. Data are presented as the mean standard deviation. **p < 0.01, ***p < 0.001. Scale bars: 100 μm.

According to the PCR and western blotting results, the differences in the expression of the multifactorial core gene TYMS were highly notable; thus, we proceeded to further analyze the expression of TYMS. Firstly, we transfected lentiviral vectors specific for TYMS knockdown into KYSE30 and KYSE150 cells and calculated the transfection efficiency (Fig. 7A). Then, we examined the cell proliferation using CCK8 cell proliferation assay. Silencing TYMS significantly reduced the proliferation abilities of KYSE30 and KYSE150 cells compared with those of the cells from the control group (Fig. 7B). The KYSE150 cell line was sensitive to lentivirus knockdown. The wound-healing assay revealed that TYMS knockdown significantly delayed wound healing (Fig. 7C-7D)(*p* < 0.001). Moreover, the invasion assay showed that TYMS knockdown significantly reduced the invasive ability of KYSE50 cells (Fig. 7E-7F). These results showed that TYMS downregulation markedly reduced the proliferation and invasion abilities of ESCC cell lines; this is consistent with the results of our aforementioned PCR and western blotting analyses.

## Discussion

ESCC is a dangerous and aggressive disease with an unfavorable prognosis [26]. After receiving radiation and chemotherapy, patients with inoperable tumors have a survival time of only 17–54 months, and patients with surgically resected EC have a 5-year survival rate of approximately 30% [27]. Mitochondria are important organelles in eukaryotes, and their dysfunction leads to many diseases [28]. However, the specific mechanisms of mitochondria and their regulatory roles in ESCC have not been investigated. Therefore, we examined the specific roles of mitochondria-related genes in ESCC and the immune microenvironment.

We identified 104 DEMAGs and screened 50 genes that were most closely correlated in the enrichment analysis. These genes were mainly enriched in respect of the regulation of the protein complex assembly, response to collagen catabolic processes, and metabolic pathways. These pathways are closely associated with mitochondria and their functions [29-31]. We identified 10 genes most strongly associated with the prognosis and survival of patients. Prognosis and survival analysis of the ten most significant core genes revealed that ALOX12 and TYMS were strongly correlated with the prognosis of ESCC. The mRNA and protein expression level of TYMS in ESCC cells was substantially higher than that in normal cells. TMYS overexpression was associated with poor survival, suggesting that the MAR model is a reliable prognostic biomarker for patients with ESCC.

ALOX12 is a lipoxygenase in the 12-LOX family, which is primarily responsible for controlling intracellular peroxyl lipid signaling and metabolism [32]. ALOX12 has been identified a biomarker and therapeutic target in many human malignancies [33]; it is closely linked to the prognosis, tumor proliferation, invasion, and metastasis of colorectal cancer, and its high expression predicts a better immunotherapy response [34]. Overexpression of ALOX12 in lung cancer increases the activities of RhoA and NF-κB, which promotes cell proliferation and migration [35]. Song *et al*. reported that ALOX12 is strongly correlated with the prognosis of patients with ESCC [36]. These results are consistent with those of our study.

TYMS is a key enzyme in the de novo thymine deoxyribonucleotide monophosphate synthesis pathway, which catalyzes uracil deoxyribonucleotides and is essential for DNA synthesis and repair [37]. Elevated expression of TYMS has been reported in pancreatic cancer and is negatively correlated with the overall survival and recurrence-free survival of patients [38]. Similarly, patients with breast cancer showing high TYMS expression have a poor prognosis [39]. According to a prior study, FOXM1-induced upregulation of TYMS promotes the progression of hepatocellular carcinoma [40]. Our model predicted that the expression of TYMS was markedly increased in ESCC and strongly correlated with the prognosis of ESCC. Furthermore, in the in vitro experiments, TYMS knockdown inhibited the proliferation of ESCC cells.

Overall, the genes used to construct the MAR model were closely related to tumor development. Prognostic models constructed using multiple genes are more comprehensive and valid than those constructed using a single gene and can be used as reliable prognostic indicators. The expression levels of these core genes differed between ESCC and normal tissues, verifying the clinical applicability of the MAR model. We also explored TME characteristics, immune cell infiltration, immunological checkpoint expression, and drug sensitivity using the MAR model.

Infiltrated immune cells are directly related to the prognosis of patients with tumors. Monocytes and macrophages release large amounts of inflammatory mediators that promote tumor growth and reduce anti-tumor immunity [41]. Monocytes and macrophages were substantially enriched in the high-risk group, with a strong positive correlation between RS and these cells. This relationship provides a favorable ecological environment for cancer growth. Regulatory cells (Tregs) prevent immune system disturbances caused by exaggerated inflammatory responses. Myeloid-derived suppressor cells facilitate tumor invasion and metastasis by stimulating the activity of immunosuppressive Tregs [42]. However, high Treg expression can cause CD8+ T cell depletion, which is a predisposing factor to various cancers [43, 44]. The high-risk group showed substantially higher levels of monocytes, macrophages, and Tregs, which may have an additive effect on tumor invasion and metastasis by preventing T cell activation and natural killer cell activity.

Monoclonal antibodies against immune checkpoint molecules have achieved considerable progress in cancer therapy. For example, CCL2 and PD-L1 have shown encouraging results in ESCC treatment [45]. The immunotherapy response rates for high-risk patients were considerably lower than those for low-risk patients. Notably, the RS and immunity or the stroma score were positively correlated. These results support that RS, as a biomarker, can predict the effectiveness of immunotherapy.

RS contributes to drug screening. In the current study, patients in the high-risk group were potentially more sensitive to AZD8055, Entinostat, GSK269962A, Nilotinib, Ruxolutinib, SB505124, and Wnt.c59, whereas patients in the low-risk group were potentially more sensitive to AZD1332, AZD7762, and Dasatinib, these results were validated using TYMS. Some drugs exert strong clinical effects. Trametinib and Dasatinib promote anti-tumor metabolic activities [46]. Treatment of hormone receptor- positive advanced breast cancer with Entinostat and exemestane has shown promising results [47]. However, side effects are associated with some of these drugs. For example, AZD7762 may lead to decreased immune system function, increased risk of infection, and unpredictable cardiotoxicity; these side effects prevent the application of these drugs in the treatment of advanced solid tumors [48]. Greater success in cancer treatment can be attained by better understanding the mechanisms of action of the drugs.

This study had some limitations. First, the sample size was small. Second, all analyses were performed with retrospective data, as prospective data from multicenter cohorts are lacking. Thus, clinical studies are required to verify the accuracy of the model.

In summary, by constructing a prediction model using a combination of multiple genes, we developed a more stable and reliable prediction model than that derived using a single gene. In addition, we incorporated mitochondria-related genes with prognostic risk function in our model, which can greatly improve predictions of the diagnosis and prognosis of patients with ESCC. Moreover, our model provides functional options for drug sensitivity prediction, immune cell infiltration, and PCR experimental validation for ESCC, thereby serving as a reliable basis for personalizing the management and treatment of patients with ESCC.

## Supplemental Materials

Supplementary data for this paper are available on-line only at http://jmb.or.kr.





## Figures and Tables

**Table 1:** Reverse transcription-polymerase chain (RT-PCR) primer sequences.

Name	Sequence (5'–3')
human-ALOX12-F	CCAAAGGGATGACATAGTGAA
human-ALOX12-R	GGTGAGGAAATGGCAGAGTT
human-TYMS-F	GGAGGAGTTGCTGTGGTT
human-TYMS-R	TCTTCTGTCGTCAGGGTTG
human-ACADVL-F	GCCAAGGCGGAATCTAAG
human-ACADVL-R	AACGGGCGTACTGGGTGT
human-MPC1-F	CCCTCTGTTGCTATTCTTT
human-MPC1-R	CTGGGCTACTTCATTTGTT
human-MRPS17-F	GGGAAGGTGATTGGGACA
human-MRPS17-R	CTGAAGGGCATCGTGAGC
human-NLRX1-F	TGTGAGGACCTGTCATCCCT
human-NLRX1-R	ACACCTGAACCGCCAACG
human-P4HA1-F	ACAAGCCCTAAGGCAACT
human-P4HA1-R	GGAAGCGAATAATACGAG
human-SLC25A32-F	TGTTCCTGGGCTGTTTGG
human-SLC25A32-R	CCGACGCCTTCTTTCCTC
human-SPTLC2-F	ATGAACATTCCTGCTCTTG
human-SPTLC2-R	TCTATCAGCTCCTTCTTGC
human-TUSC3-F	AGTTCCAGACGCTCAATC
human-TUSC3-R	CCCTCATCATAGTCCACC

**Table 2:** Detailed information on ALOX12 and TYMS.

Gene symbol	Gene ID	Full name	Function of the encoded protein
ALOX12	245	arachidonate 12-lipoxygenase, 12R-type	The 12-HETE regulated by ALOX12 plays important roles in the body, including regulating precursor synthesis, cell proliferation, and inflammation response
TYMS	7298	thymidylate synthetase	The thymidylate synthetase encoded by the TYMS gene plays an important role in regulating nucleotide synthesis, cell proliferation, and DNA stability.

**Table 3:** Detailed information on the top 10 sensitive drugs.

Drug name	Drug target	Target pathway	Introduction
AZD1332	NTRK1, NTRK2, NTRK3	RTK signaling	AZD1332 is a potent, selective, ATP-competitive neurotrophic tyrosine kinase receptor.
AZD7762	CHEK1, CHEK2	Cell cycle	AZD-7762 (AZD7762) is a potent and selective inhibitor of Chk1/2，enhances the radiosensitivity of mutated p53 tumor cell lines and HT29 xenografts.
Dasatinib	ABL,SRC,Ephrins, PDGFR, KIT	Other	Dasatinib inhibits tumor growth and spread by inhibiting the activity of BCR-ABL and other kinases, as well as interfering with aberrant signaling pathways, and is an important drug used in the treatment of leukemia and other malignancies.
AZD8055	MTORC1, MTORC2	PI3K/MTOR signaling	AZD8055, as a bi-directional mTOR inhibitor, has the potential to be used as a therapeutic agent for the treatment of tumors by inhibiting the growth, live and metastatic ability of tumor cells through the inhibition of both components of the mTOR signaling pathway.
Entinostat	HDAC1, HDAC3	Chromatin histone acetylation	Entinostat, as an HDAC inhibitor, mainly works by regulating chromatin structure and gene transcription, affecting the cell cycle, DNA repair, etc. thereby inhibiting tumor cell proliferation and growth.
GSK269962A	ROCK1, ROCK2	Cytoskeleton	GSK269962A represents a novel class of ROCK inhibitors that have profound effects in the vasculature and may enable us to further evaluate the potential beneficial effects of ROCK inhibition in animal models of cardiovascular as well as other chronic diseases.
Nilotinib	ABL	ABL signaling	Nilotinib is a drug used to treat chronic myeloid leukemia and certain acute lymphoblastic leukemias by inhibiting the activity of BCR-ABL and other related tyrosine kinases, interfering with aberrant signaling pathways, and inhibiting the proliferation and inducing the apoptosis of leukemia cells.
Ruxolutinib	JAK 1?TYK 2	JAK signaling	Ruxolitinib, as a JAK inhibitor, interferes with cytokine signaling pathways and inhibits the activation of aberrant signaling pathways by inhibiting the activity of kinases such as JAK1 and JAK2, and has immunomodulatory effects.
SB505124	TGFBR1, ACVR1B, ACVR1C	RTK signaling	SB505124 acts as an inhibitor of the TGF-β signaling pathway, and its main role is to block TGF-β signaling by inhibiting TGF-β receptor activity, inhibiting the proliferation, migratory and invasive ability of tumor cells.
Wnt.c59	PORCN	Wnt signaling	By inhibiting the Wnt signaling pathway, Wnt.c59 can play a regulatory role in a variety of cells and tissues.
