# Supplementary figures and images for: Construction of a Novel Mitochondria-Associated Gene Model for Assessing ESCC Immune Microenvironment and Predicting Survival
Source: J Microbiol Biotechnol. 2024 Feb 22;34(5):1164–77. doi: 10.4014/jmb.2310.10052 (PMC11180922; doi:10.4014/jmb.2310.10052)

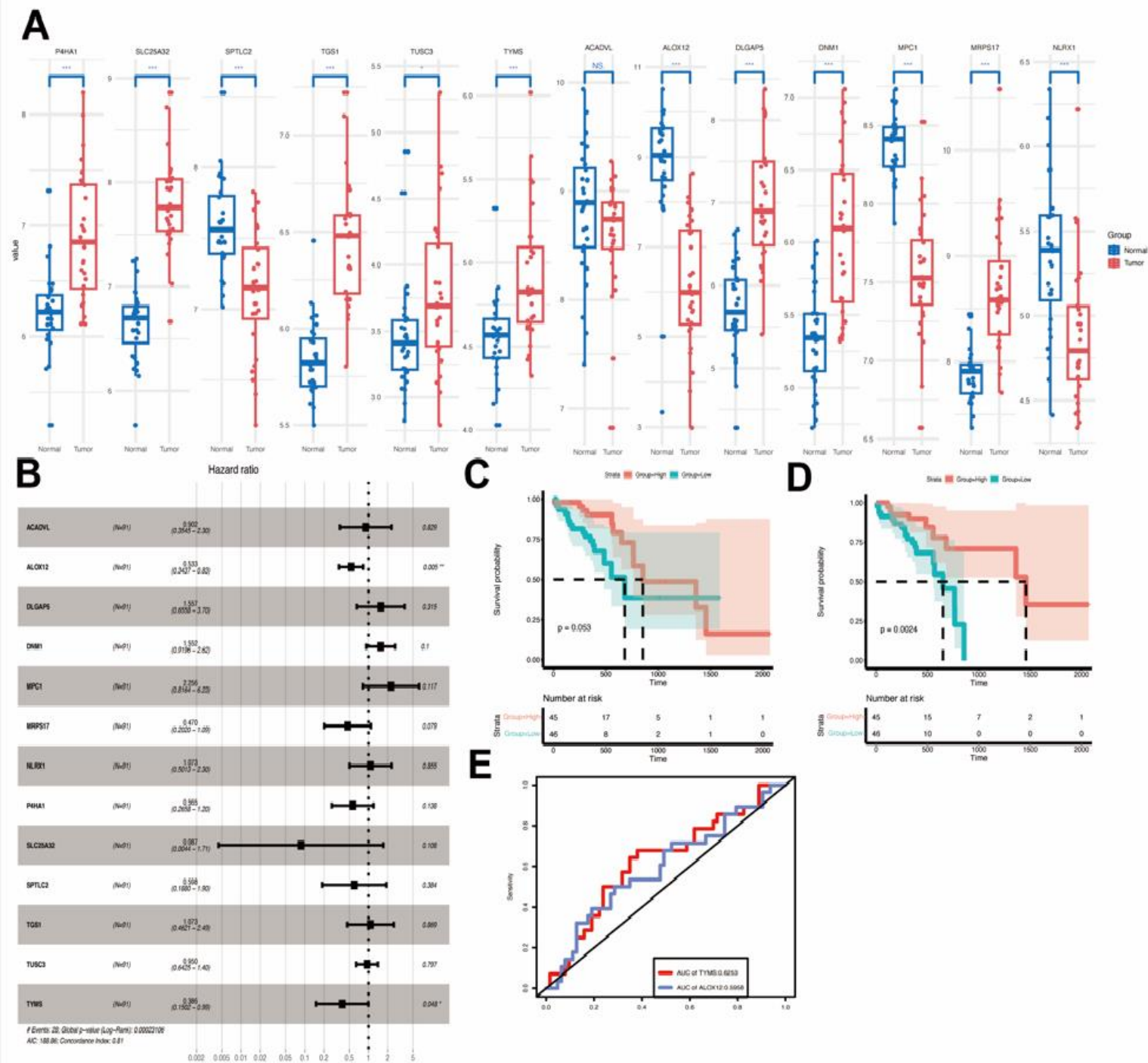

Fig. S1

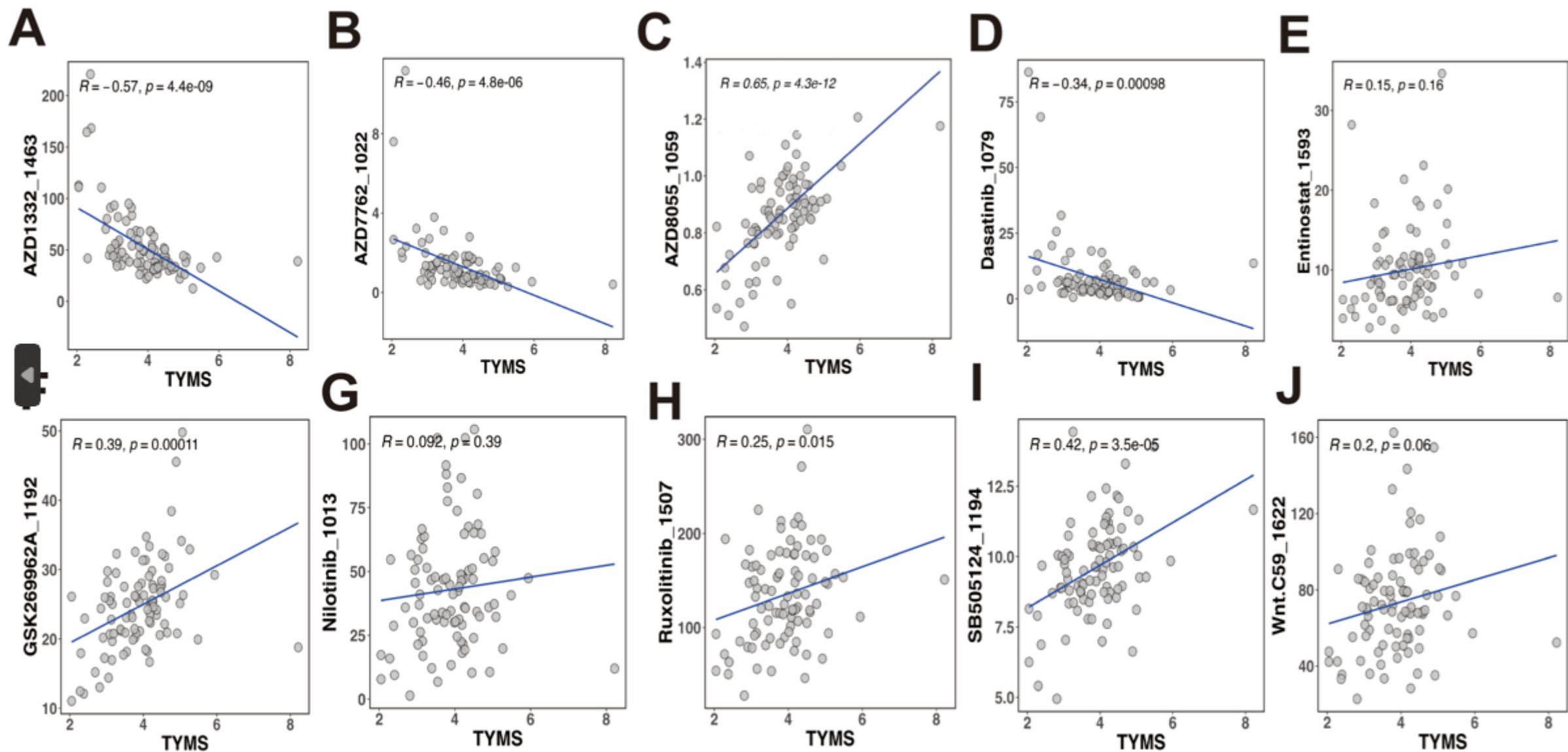

Fig. S2

Supplement: Supplementary file 1 [file jmb-34-5-1164-supple1.pdf]
