# Supplementary material for: Construction of a Novel Mitochondria-Associated Gene Model for Assessing ESCC Immune Microenvironment and Predicting Survival
Source: J Microbiol Biotechnol. 2024 Feb 22;34(5):1164–77. doi: 10.4014/jmb.2310.10052 (PMC11180922; doi:10.4014/jmb.2310.10052)
Supplement: Supplementary file 2 [file jmb-34-5-1164-supple2.pdf]

Supplementary Tables

Table S1. Clinical information.

	Alive<br>*N=63*	Dead<br>*N=28*	p.overall
Age:			0.849
>65	50 (79.4%)	21 (75.0%)	
<65	13 (20.6%)	7 (25.0%)	
Gender:			0.212
Female	12 (19.0%)	2 (7.14%)	
Male	51 (81.0%)	26 (92.9%)	
Tumor_stage:			0.117
I/II	56 (88.9%)	21 (75.0%)	
III/IV	7 (11.1%)	7 (25.0%)	
T_stage:			1.000
T1/T2	27 (42.9%)	12 (42.9%)	
T3/T4	36 (57.1%)	16 (57.1%)	
N_stage:			0.079
N0	41 (65.1%)	12 (42.9%)	
N1/N2/N3	22 (34.9%)	16 (57.1%)	
Alcohol_history:			0.140
No	20 (31.7%)	4 (14.3%)	
Yes	41 (65.1%)	24 (85.7%)	
Not reported	2 (3.17%)	0 (0.00%)	
RiskScore:			0.003
Low	39 (61.9%)	7 (25.0%)	
High	24 (38.1%)	21 (75.0%)	

Table S2. Two thousand and thirty mitochondria-related genes.

Mitochondrial Genes (n=2030)	Mitochondria-related GSEA Gene Sets(n=163)
ABCF2	GOBP_ANTEROGRADE_AXONAL_TRANSPORT_OF_MITOCHONDRION
ACBD6	GOBP_AUTOPHAGY_OF_MITOCHONDRION
ACBD7	GOBP_AXONAL_TRANSPORT_OF_MITOCHONDRION
ACE	GOBP_CALCIUM_IMPORT_INTO_THE_MITOCHONDRION
ACE2	GOBP_ESTABLISHMENT_OF_MITOCHONDRION_LOCALIZATION
ACOT1	GOBP_MAINTENANCE_OF_PROTEIN_LOCATION_IN_MITOCHONDRION
ACOT12	GOBP_MITOCHONDRION_DISTRIBUTION
ADORA2A	GOBP_MITOCHONDRION_LOCALIZATION
AGTR1	GOBP_MITOCHONDRION_MORPHOGENESIS
AGTR2	GOBP_MITOCHONDRION_ORGANIZATION
AKT2	GOBP_NEGATIVE_REGULATION_OF_AUTOPHAGY_OF_MITOCHONDRION
ALOX12	GOBP_NEGATIVE_REGULATION_OF_ESTABLISHMENT_OF_PROTEIN_LOCALIZATION_TO_MITOCHONDRION
ARL6IP5	GOBP_NEGATIVE_REGULATION_OF_MITOCHONDRION_ORGANIZATION
CAMK4	GOBP_NEGATIVE_REGULATION_OF_PROTEIN_TARGETING_TO_MITOCHONDRION
CARM1	GOBP_POSITIVE_REGULATION_OF_AUTOPHAGY_OF_MITOCHONDRION
CASP7	GOBP_POSITIVE_REGULATION_OF_ESTABLISHMENT_OF_PROTEIN_LOCALIZATION_TO_MITOCHONDRION
CAV3	GOBP_POSITIVE_REGULATION_OF_PROTEIN_TARGETING_TO_MITOCHONDRION
CCL27	GOBP_PROTEIN_LOCALIZATION_TO_MITOCHONDRION
CGAS	GOBP_PROTEIN_PROCESSING_INVOLVED_IN_PROTEIN_TARGETING_TO_MITOCHONDRION
CHD9	GOBP_PROTEIN_TARGETING_TO_MITOCHONDRION
CHKB	GOBP_REGULATION_OF_AUTOPHAGY_OF_MITOCHONDRION
CREBBP	GOBP_REGULATION_OF_AUTOPHAGY_OF_MITOCHONDRION_IN_RESPONSE_TO_MITOCHONDRIAL_DEPOLARI
CRTC1	GOBP_REGULATION_OF_ESTABLISHMENT_OF_PROTEIN_LOCALIZATION_TO_MITOCHONDRION
CRTC2	GOBP_REGULATION_OF_MITOCHONDRION_ORGANIZATION
CRTC3	GOBP_REGULATION_OF_PROTEIN_TARGETING_TO_MITOCHONDRION
CTSL	GOBP_RNA_IMPORT_INTO_MITOCHONDRION
DDX58	GOCC_MITOCHONDRION
ELAVL1	GOMF_MITOCHONDRION_TARGETING_SEQUENCE_BINDING
G6PD	HP_ABNORMALITY_OF_THE_MITOCHONDRION
GABPB1-IT1	REACTOME_RRNA_MODIFICATION_IN_THE_MITOCHONDRION
GOT1	REACTOME_RRNA_PROCESSING_IN_THE_MITOCHONDRION
HDAC3	REACTOME_TRNA_MODIFICATION_IN_THE_MITOCHONDRION
HELZ2	REACTOME_TRNA_PROCESSING_IN_THE_MITOCHONDRION
HMGA1	GOBP_APOPTOTIC_MITOCHONDRIAL_CHANGES
HSD17B12	GOBP_ESTABLISHMENT_OF_PROTEIN_LOCALIZATION_TO_MITOCHONDRIAL_MEMBRANE
IFIH1	GOBP_INNER_MITOCHONDRIAL_MEMBRANE_ORGANIZATION
IL1B	GOBP_MITOCHONDRIAL_ACETYL_COA_BIOSYNTHETIC_PROCESS_FROM_PYRUVATE
IL6	GOBP_MITOCHONDRIAL_ADP_TRANSMEMBRANE_TRANSPORT
IRF7	GOBP_MITOCHONDRIAL_ATP_SYNTHESIS_COUPLED_PROTON_TRANSPORT
MAPK11	GOBP_MITOCHONDRIAL_CALCIUM_ION_HOMEOSTASIS
MDH1	GOBP_MITOCHONDRIAL_CALCIUM_ION_TRANSMEMBRANE_TRANSPORT
MED1	GOBP_MITOCHONDRIAL_CYTOCHROME_C_OXIDASE_ASSEMBLY
MEF2C	GOBP_MITOCHONDRIAL_DEPOLARIZATION

Table S3. Univariate and multivariate Cox regression analyses of various prognostic parameters in patients with esophageal squamous cell carcinoma.

haracteristics	Total(N)	Univariate analysis			Multivariate analysis	
		Hazard ratio (95% CI)	P value		Hazard ratio (95% CI)	P value
Age	91					
<=60	59	Reference				
>60	32	1.538 (0.690 - 3.431)	0.293			
Gender	91					
female	14	Reference			Reference	
male	77	4.885 (1.120 - 21.305)	**0.035**		5.928 (1.152 - 30.503)	**0.033**
umor_stage	91					
StageI	7	Reference			Reference	
StageII	54	1.013 (0.222 - 4.613)	0.987		0.566 (0.110 - 2.922)	0.497
StageIII	26	2.634 (0.576 - 12.049)	0.212		0.589 (0.086 - 4.023)	0.589
StageIV	4	3.090 (0.502 - 19.009)	0.223		1.175 (0.133 - 10.367)	0.884
T_stage	91					
T1	8	Reference				
T2	31	1.114 (0.293 - 4.238)	0.874			
T3	48	1.136 (0.320 - 4.029)	0.844			
T4	4	4.003 (0.769 - 20.839)	0.099			
N_stage	91					
N0	53	Reference			Reference	
N1	28	1.754 (0.755 - 4.076)	0.192		1.199 (0.389 - 3.698)	0.752
N2	7	3.400 (1.083 - 10.676)	**0.036**		3.663 (0.782 - 17.163)	0.099
N3	3	4.783 (1.036 - 22.086)	**0.045**		2.220 (0.361 - 13.652)	0.390
cohol_history	91					
Yes	65	Reference				
No	24	0.532 (0.183 - 1.548)	0.247			
ot Reported	2	0.000 (0.000 - Inf)	0.998			
Score	91					
Low	46	Reference			Reference	
